# State Care of Epileptics

**Published:** 1896-03

**Authors:** F. S. White, C. O. Matthews, James Orr

**Affiliations:** Chairman; Terrell, Texas; Committee; Terrell, Texas; Committee; Terrell, Texas


### State Care of Epileptics.

TERRELL, TEXAS, February 13, 1896.

*Editors Texas Medical Journal:*

The following resolutions were passed by the Terrell Medical Society February 4, 1896, and the president appointed the undersigned on that committee:

WHEREAS, An effort is being made by the medical profession of Texas to induce the next legislature to enact a just law regulating the practice of medicine; and

WHEREAS, The provision for taking care of chronic insane, epileptics, and the habitues of alcohol, opium, cocaine and other narcotics, is wholly inadequate to meet the demand, and that hundreds of these unfortunate cases, to their detriment, and contrary to the dictates of humanity, are now languishing in jails and poor-houses throughout the State; therefore, be it

*Resolved*, That this society deprecates and condemns this condition of affairs, and urges that the State, at the earliest possible moment, declare all these unfortunate people her wards and make provision for their care and treatment; be it further

*Resolved*, That the president of this society shall appoint a committee whose duty it shall be to invite the co-operation of all other local medical societies, as well as individual members of the profession, in aiding us in presenting these matters to the State Medical Association at its next meeting, and through it to the legislature.

It is our purpose to endeavor to organize the entire medical profession of Texas into a working body and try once more to remedy these existing evils. This can only be accomplished through persistent work and concert of action. We hope every physician and philanthropic citizen of Texas will give us their support in this matter. We beg to urge yon in the name of humanity to forcibly present these matters to the men who will represent you in the next legislature. The great reason why no law has been passed heretofore to properly regulate the practice of medicine in this State is because the members of the legislature have not had the matter intelligently presented to them at home. The legislators, as a rule, are men who desire to enact just laws and remedy existing evils. The other matters mentioned are of very great importance. There are to-day, at a very low estimate, between 1000 and 1500 insane in this State outside of the asylums. A large number of these are confined in filthy and unhealthy jails and poor houses, mixed, on account of misfortune and disease, with all grades of criminals.

Epilepsy is one of most deplorable and incurable conditions that afflicts mankind, and its victims should have special provision made for their treatment and care. The unfortunate habitues of alcohol, morphine, cocaine, etc., in the light of scientific research are considered the victims of disease and capable of being cured.

The increasing numbers of these cases demands that something be done to prevent the formation of these habits, and to relieve and reform those already afflicted. To remedy these evils, we suggest that the State erect an asylum on the cottage system, capable of accommodating all the insane in the State, especially the chronic insane; and that attached to this there be a department exclusively for epileptics and one for inebriates and habitues of narcotics.

F. S. WHITE, M. D.,
Chairman;
C. O. MATTHEWS, M. D.,
JAMES ORR, M. D.,
Committee.

